# The effect of corticosteroid versus platelet-rich plasma injection therapies for the management of lateral epicondylitis: A systematic review

**DOI:** 10.1051/sicotj/2017062

**Published:** 2018-03-21

**Authors:** Walid Ben-Nafa, Wendy Munro

**Affiliations:** 1 The University of Salford, Salford UK; 2 Trauma and Orthopaedics department, Central Manchester University Hospitals NHS Foundation Trust UK

**Keywords:** Tennis elbow, Lateral epicondylitis, Epicondylopathy, Epicondylalgia Corticosteroid, Steroid injections, Platelet-rich plasma, PRP injections

## Abstract

*Introduction*: Lateral epicondylitis is a common musculoskeletal disorder of the upper limb. Corticosteroid injection has been widely used as a major mode of treatment. However, better understanding of the pathophysiology of the disease led to a major change in treating the disease, with new options including platelet-rich plasma (PRP) are currently used.

*Objectives/research aim*: To systematically evaluate the effect of corticosteroid versus PRP injections for the treatment of LE.

*Hypothesis*: PRP injections provide longer-term therapeutic effect and less rate of complications compared to corticosteroid injection.

*Level of evidence*: Level 2 evidence (4 included studies are of level 1 evidence, 1 study of level 2 evidence).

*Design*: Systematic Review (according to PRISMA guidelines).

*Methods*: Eleven databases used to search for relevant primary studies comparing the effects of corticosteroid and PRP injections for the treatment of LE. Quality appraisal of studies performed using Cochrane Handbook for Systematic Reviews of Interventions Version 5.1.0, CASP Randomised Controlled Trial Checklist, and SIGN Methodology Checklist 2.

*Results*: 732 papers were identified. Five randomised controlled trials (250 Patients) met the inclusion criteria. *Clinical findings*: Corticosteroid injections provided rapid symptomatic improvement with maximum effect at 6/8/8 weeks before symptoms recurrence, whereas PRP showed slower ongoing improvements up to 24/52/104 weeks(3 studies). Corticosteroid showed more rapid symptomatic improvement of symptoms compared to PRP up to the study end-point of 3 months(1 study). Comparable therapeutic effects of corticosteroid and PRP were observed at 6 weeks(1 study). *Ultrasonographic Findings*: (1) Doppler activity decreased more significantly in patients who received corticosteroid compared to PRP. (2) Reduced tendon thickness and more patients with cortical erosion noted in corticosteroid group whereas increased tendon thickness and less number of patients with common extensor tendon tears noted in PRP group. (3) Fewer patients reported Probe-induced tenderness and oedema in the common extensor tendon in both corticosteroid and PRP groups (2 studies).

*Conclusion*: Corticosteroid injections provide rapid therapeutic effect in the short-term with recurrence of symptoms afterwards, compared to the relatively slower but longer-term effect of platelet-rich plasma.

##  Introduction

Tennis elbow or lateral epicondylitis (LE), is one of the most common and painful musculo-skeletal conditions, which has a significant impact on the healthcare industry and society in general [1,2]. Lateral epicondylitis is a common term used to describe a group of symptoms including pain and tenderness over the origin of extensor muscles of the wrist and fingers [3,4]. With a prevalence rate of more than 1% among the general population, and with a slight predominance among females [5,6] the disease mostly affects people aged between 35–50 years, who have a history of repetitive activities involving the upper limb [7,8]. While the exact pathophysiology behind the condition is not yet clear, and despite the presence of inflammatory cells locally, there is a strong argument that LE can be regarded as a degenerative process caused by muscle overuse, with subsequent tendinosis, micro-trauma and tear of the extensor carpi radialis brevis tendon [9,10].

The relatively large number of options currently available for the treatment of lateral epicondylitis [11,12], could be attributed to the scarce evidence available about the disease aetiology and the lack of agreement about a definitive treatment for the condition. Corticosteroid injection has been considered a major treatment option for LE [9]. As corticosteroid mainly aims to reduce inflammation, it is apparently questionable whether it has any possible long-term healing potential with the disease's degenerative changes [10]. Besides the known local and systemic side effects associated with its use [11,13–16], some researchers believe that corticosteroid injections may even delay the natural recovery expected with watchful waiting or other management options [17], in addition to the high rate of symptoms recurrence noted with its use.

With recent histological findings showing that tendinosis is not an acute inflammatory condition but rather a failure of normal tendon repair [18,19] researchers started to focus on the function of biologics in treating LE [20], including Platelet-rich Plasma (PRP) injection [21]. Firstly introduced by Whitman, Berry, & Green [22], PRP has a 3–5 fold increase of platelet concentration compared to whole blood levels [23], and includes many growth factors essential for bone-to-tendon healing, as well as other vascular, epidermal and connective tissue growth factors [24]. Different laboratory studies showed enhanced tenocyte proliferation after injection with platelet-released growth factors [25,26]. However, and with the insufficient evidence available to support its clinical use [27], PRP injections continues to be debated in the literature, especially with the lack of its comparable rapid therapeutic effect compared to other pharmacological treatments [28,29].

Although a number of randomised controlled trials compared the effect of corticosteroid and PRP injection for treating LE, an up-to-date systematic review of these primary studies is lacking within the literature. While some reviews [17,30] assessed the effect of various injection therapies for LE, none of these studies investigated the effect of PRP injection as a comparative therapy for the treatment of LE. On the other hand, and while a number of systematic reviews [31–33] focused on the effect of PRP injections, none of these compared the outcome of this injection therapy with other injection modalities, including corticosteroid injections. Some reviews and meta-analyses recently included more primary studies about the effect of PRP and steroid injections [34–36], but again, none of these reviews focused or specifically compared the effect of steroid and PRP injection therapies. This attributed to the fact that these reviews were aiming at collectively comparing the effects of different injection therapies to obtain a general conclusion about their effect in LE. As a result, this has hindered for a conclusion to be drawn about the effects peculiar to steroid and PRP injections, leaving a gap in the literature, with inadequate knowledge available to inform the relevant clinical practices. In this systematic review, we aim to systematically accumulate the evidence available from the relevant primary studies about the effect of these two treatments. We hypothesise that PRP injection provides longer-term therapeutic effect and less rate of complications compared to corticosteroid injection.

##  Methodology

**Study selection criteria** Although the main focus was on RCTs, all interventional studies were eligible for inclusion in this study. The search strategy of this review was structured using Population, Intervention, Comparison, Outcome framework (PICO). Free-text searching and Medical Subject Headings were also considered.

**Figure 1:**
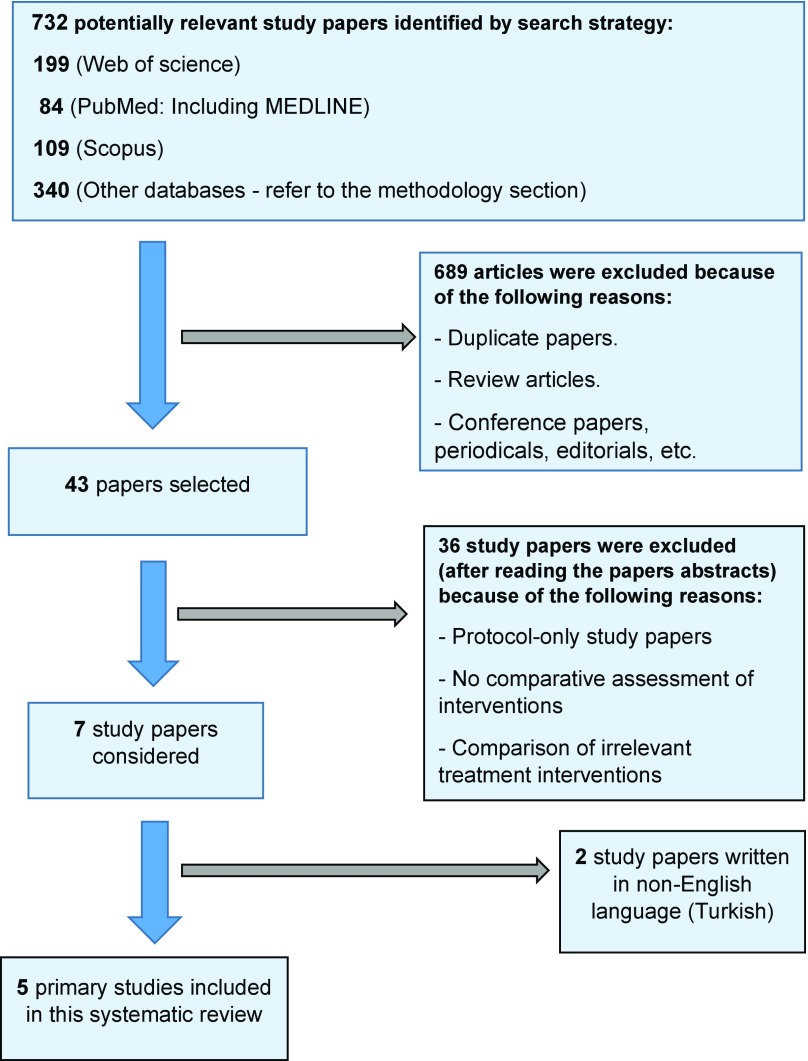
PRISMA Flow Chart showing the studies' selection process which led to the inclusion of the five study.

**Search strategy** The search strategy was conducted and reported in accordance with PRISMA guidelines [37] and Cochrane Collaboration [38] whenever possible. It also adhered to the published guidance used when undertaking reviews in healthcare [39]. Eleven databases were systematically searched, namely, Web of science, Scopus, PubMed (MedLine), ScienceDirect, CINAHL, EMBASE, Ovid, NICE, Physiotherapy Evidence Database, Cochrane Library and ClinicalTrials database (Figure 1). As we aimed to search for only the recent and relevant evidence, only papers published from 2005 onwards were accessed, and hand-searched for their bibliographic references to look for any relevant references. The search was also enhanced using forward citation searching strategy, with searching by author and study title was also considered using Web of Science Service. Cochrane Database of Systematic Reviews, Cochrane Central Register of Controlled Trials, and database of abstracts of reviews of effectiveness were also consulted. Potentially relevant studies, which are labelled as ongoing studies, were searched for using International Clinical Trials Registry Platform, UK Clinical Research Network Study Portfolio, US National Library of Medicine Database of Clinical Trials and metaRegister of Controlled Trials. Database bias was eliminated as possible, by reviewing journals which were not indexed in major bibliographic databases. Finally, potentially relevant studies in the grey literature was also considered to identify any unpublished research or research produced outside the traditional distribution channels.

Only Papers published in English language were included in this review, although it was aimed initially to consider papers written in non-English languages too, including two papers in Turkish language. Nevertheless, and as the accuracy of quality assessment and data extraction could be affected when translating non-English papers, it was finally decided to restrict the work on papers written in English language. This was also supported by the evidence that language restriction does not appear to change the results of systematic reviews conducted on RCTs of conventional interventions [40].

Some of the experts in the field pertinent to this review, including some of the included studies' authors, were contacted to inquire about any ongoing or unpublished relevant studies. No relevant studies were declared by the authors.

The process of inclusion of relevant studies was independently conducted by the two authors, and consensus has been reached between the two reviewers with no need for a third opinion. Exclusion criteria included old studies conducted before 2005, study population with elbow pain/pathologies other than LE, and injection materials other than corticosteroid or PRP, including autologus blood and autologous conditioned plasma injections, which were excluded due to their irrelevancy.

The data extraction process was performed before assessing the quality of the studies, to enhance the objectivity in collecting data regardless of the quality. The process of data extraction was carried out independently by the two reviewers as well. A pilot data extraction form [41] was created and tested by the two reviewers before embarking on the data extraction process, to test the comprehensiveness and objectivity of the extraction form.

To objectively illustrate all the potential biases of the studies in a comparative style, a table has been compiled to evaluate the included studies against different checklist elements used to assess potential biases in RCTs [41–44]. The reviewers independently appraise and eventually agreed on the quality of the included studies (Table 1).

**Table 1:**
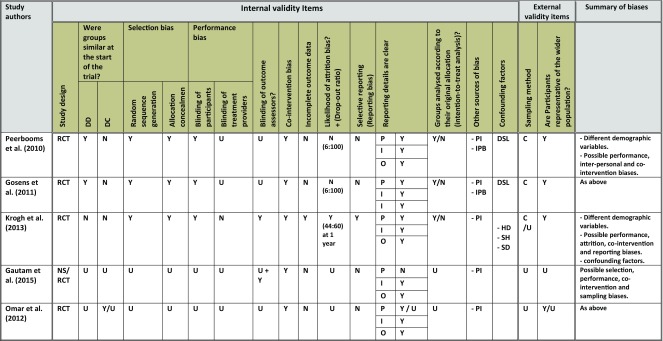
The result of quality assessment of the included studies.

Y: Yes; N: No; C: Consecutive sampling; U: Unclear; RCT: Randomised controlled trials; NS: Not stated; DD: Demographic details; DC: Disease characteristics; P: Population; I: Intervention; O: Outcomes; OM: Outcome measures; DSL: Different study locations; HD: Hand dominance; SH: Smoking history; SD: Symptoms durations; PI: Previous interventions; IPB: Inter-personal bias.

##  Results

Eleven electronic databases were searched up to February 2017 without language, publication, or study-designs restrictions (Figure 1). Out of 732 papers identified in the body of literature, 43 study papers were considered after excluding duplicate papers, review articles, conference papers and other irrelevant articles. Five studies [29,45–48], including 250 patients, were finally considered in this systematic review After reading full-text papers. The quality appraisal of the included studies is summarised in Table 1. In addition, Studies' characteristics, demographic details and details of treatment preparations have also been listed (Tables 2–4).

**Analytic data of the included studies** With careful observation of the studies' outcome results (Tables 5–7), it can be concluded that there was a general trend by most of the studies showing that PRP has slower but longer-term clinical effect with no recurrence of symptoms over the follow-up periods included. This was almost the opposite with the use of corticosteroid injections.

**Table 2:**
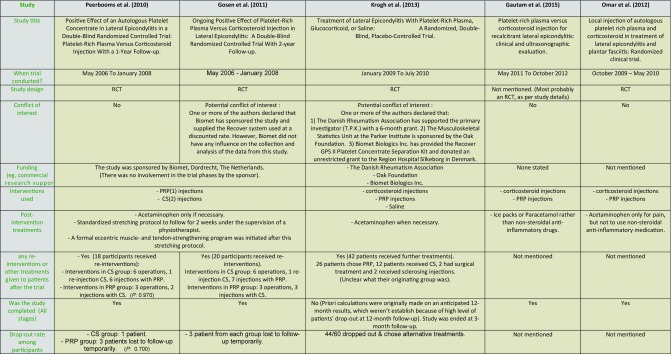
Characteristics of the included studies.

**Table 3:**
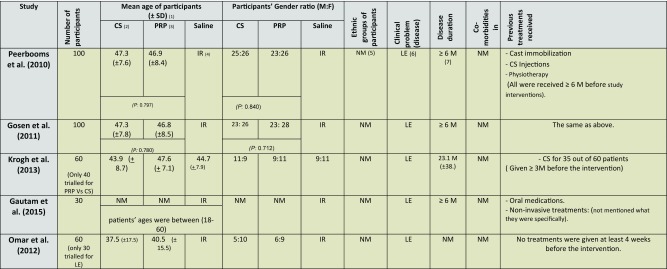
Population characteristics of the review studies.

(1) SD: Standard deviation. (2) CS: corticosteroid. (3) PRP: platelet-rich plasma. (4) IR: Irrelevant. (5) NM: Not mentioned. (6) LE: Lateral Epicondylitis. (7) M: Month. (P: P value)

**Table 4:**
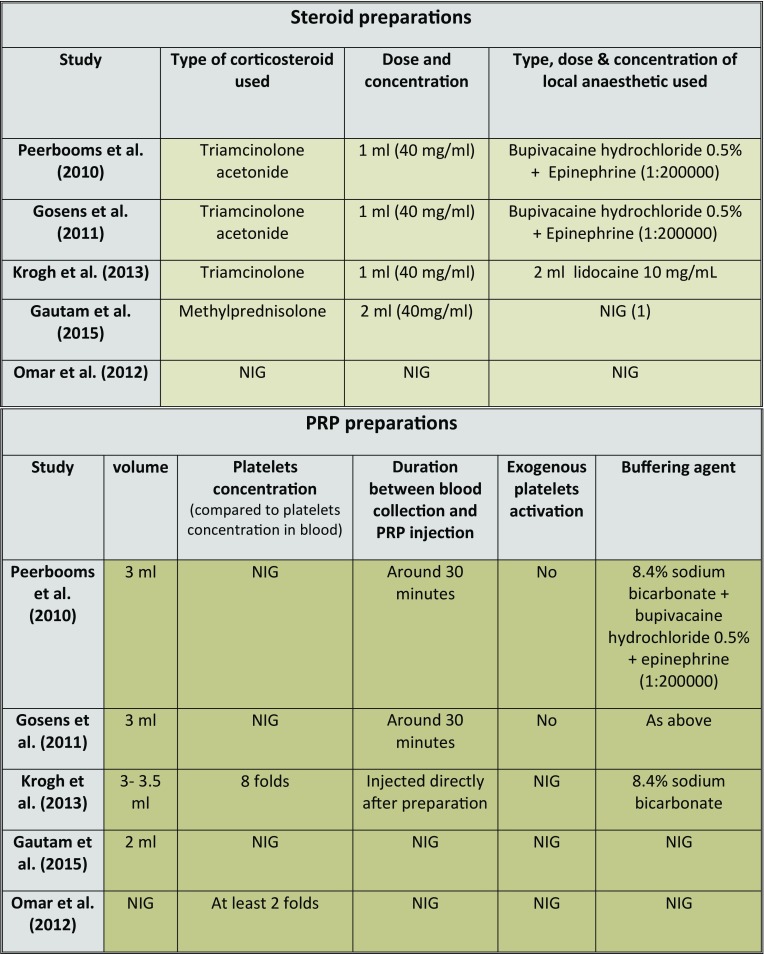
Details of steroid and PRP preparations used by the included studies.

(1) NIG: No information given

**Table 5:**
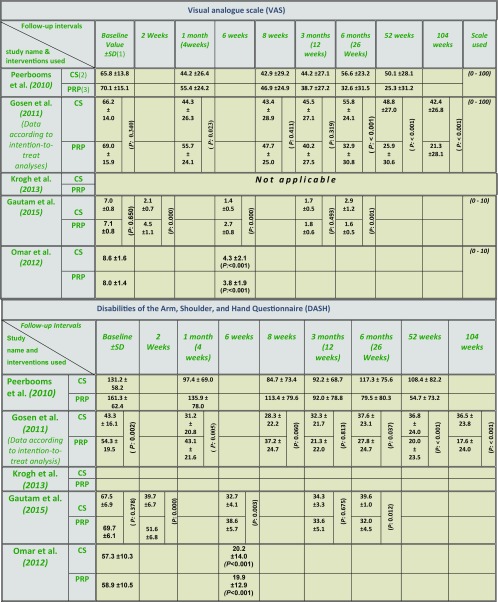
Values for Visual analogue scale (VAS) & Disabilities of the Arm, Shoulder and Hand (DASH) as reported by the included studies. (1) SD: Standard deviation; (2) CS: Corticosteroid; (3) PRP: Platelet-rich plasma. (*P*: *P* value).

(1) SE: Standard error. (2) CS: Corticosteroid. (3) PRP: Platelet-rich plasma.

**Table 6:**
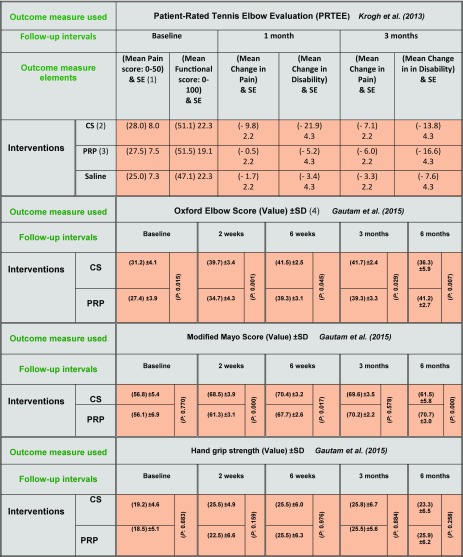
Outcome values for Patient-Rated Tennis Elbow Evaluation (PRTEE), Oxford elbow score, Modified Mayo score and Hand grip strength at different follow-up intervals.

(1) SE: Standard error. (2) CS: Corticosteroid. (3) PRP: Platelet-rich plasma. (4) SD: Standard deviation. (P:P value)

**Table 7:**
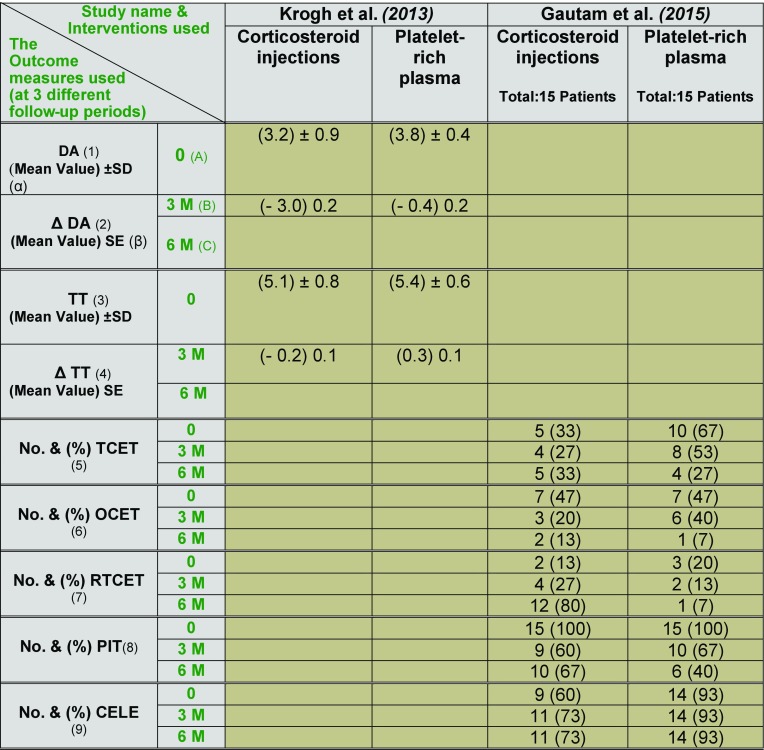
Ultrasonographic outcome data reported by 2 studies.

(1) DA: Doppler activity (Grades 0–4); (2) Δ DA: Change in Doppler activity; (3) TT: Thickness of common extensor tendon (in millimetres); (4) Δ TT: Change in Thickness of common extensor tendon; (5) No. and (%) TCET: Number and percentage of patients who had tears in the common extensor tendon; (6) No. and (%) OCET: Number and percentage of patients who had oedema in the common extensor tendon; (7) No. and (%) RTCET: Number and percentage of patients who had reduced thickness of the common extensor tendon; (8) No. and (%) PIT: Number and percentage of patients who had probe-induced tenderness; (9) No. and (%) CELE: Cortical erosion at the lateral epicondyle. (α) SD: Standard deviation; (β) SE: Standard error. (A) 0: baseline assessment; (B) 3M: 3 month; (C) 6M: 6 months.

**Safety of interventional materials used** Besides some of the known local adverse effect [49,50], no other complications or systematic effects were reported (Table 8). Except for one study [29], The lack of details regarding the rate/number of complications incidence among the participants prevents any quantitative analysis to assess the prevalence of these complications among studies' participants.

**Table 8:**
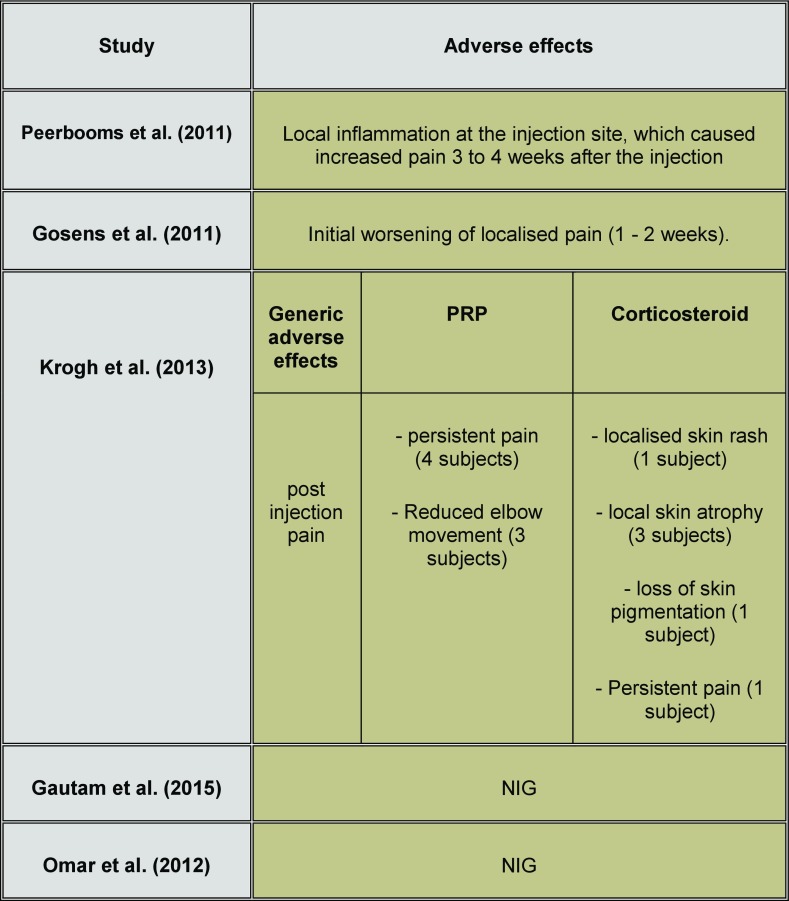
Adverse effects of injection therapies as reported by the review studies.

(NIG: No information given)

##  Discussion

Five primary studies were included in this systematic review (Table 2). All these studies were high in the hierarchy of evidence and followed the randomised controlled trials (RCTs) design [51]. Two of these studies [45,46] were merely a two-stage trial rather than two distinctive studies. Initially, Peerbooms and colleagues investigated the effect of corticosteroid versus PRP injections, with an end-point assessment at 1 year, which was followed by an assessment of the longer-term effect of the two interventions at 2 years, by Gosens and co-researchers. However, and as the two studies were not conducted by exactly the same researchers, and with two distinctive articles with study titles, it appears that the authors in the first study did not originally plan to measure the patient's outcomes at 2 years. This have entailed some changes in the studies settings, including the use of two different variants of one of the main outcome measures used (DASH). This was declared by one of the authors who stated that all DASH items were summed in the first study, yet the core elements only were used to calculate the DASH score in the second study. Secondly, the two studies were conducted at two teaching hospitals, with no details about which patients were assessed in each hospital, and how much the hospital settings were similar, and thus more details could have eliminated any potential bias.

While consecutive sampling was adopted by two studies [45,46], no details provided by the other studies [29,47,48]. Population's details, which are important to assess the external validity, varied widely among the included studies (Table 3). Age and gender ratios of participants were generally representative of the population, although no demographic data were provided by one study [47]. Also, and while Krogh and co-researchers (2013) [29] provided the most extensive details of patient's demographic data including smoking history, the percentage of patients who were current smokers varied widely among the three study arms, which ranged between 10 % in saline group, to around one-third in PRP and glucocorticoid groups. With the known correlation between smoking and delayed tissue healing [52,53], the variation in this demographic element (smoking) can be considered as a confounding factor especially if we observe the comparable therapeutic effect noted in saline (placebo) group compared to corticosteroid and PRP groups.

The randomisation process and allocation of study population was not reported by two studies [47,48], which entails a potentiality of selection bias, and questions the quality of these two high-level evidence RCTs. Additionally, the lack of blinding of outcome assessors was one of the main limitations faced by most of the included studies. Krogh and co-researchers [29] were the best who reported these elements. Peerbooms and co-researchers [45] only reported the blinding of patients to treatment interventions, Gautam and colleagues [47] only reported the blinding of ultrasound operator(s), while the study by Omar and co-researchers [48] lacked the information about all the elements of the blinding process.

Treatments received by the participants before starting the trials is another important element. Although most studies reported that patients received no treatments in the last 3–6 months before the interventions, there is still a potential long-term effect of any treatments given before that, this in addition to the possible treatment-treatment interactions after receiving the new interventional treatments. This might be of real significance if we noted, for instance, that steroid injection was received by as high as 58 % of patients received corticosteroid injections in the trial by Krogh and co-researchers [29]. Additionally, and with the considerable variation of the disease duration before the treatment interventions, it is unclear how far this kind of heterogeneity has a role when assessing the comparative effect of the treatments given.

**Injection materials used** While the use of corticosteroid as a control injection can be acceptable to compare the effect of PRP, the use of such medicinal therapeutic agent itself may undermine the accurate assessment of the effect of PRP as a treatment option. For example, the probable deleterious effects of corticosteroid [17] may falsely exaggerate the beneficial effect of PRP in comparison. Additionally, and although Krogh and co-researchers avoided this potentially confounding element by using saline as a placebo injection, the saline material itself could be considered more than an inactive comparator substance, with its possible beneficial/harmful effects as well.

The use of different types, doses and concentrations of the interventional steroid preparations (Table 4) should be noted, especially with the known difference of the therapeutic effect of these preparations [55,56]. Additionally, the combined use of local anaesthetics with steroid preparations in some studies [29,45] can be a source of bias as well, as these anaesthetic agents add volume to the injectate and help distributing the corticosteroid substance within the affected tissues [57], with possible changes in the effect of corticosteroid injections. This kind of heterogeneity can be seen more profoundly with the use of PRP injections, where different preparation methods, various commercial centrifugation/separation systems as well as different concentrations of platelets, growth factors and other blood products in the PRP preparations were used [58–63] (Table 4). With no ideal concentrations of platelets and growth factors in the PRP preparations has been established yet, these sources of variability were clearly observed in the included studies. This in addition to technical details including centrifugation speed, buffering agents used to achieve physiological pH, and the time needed for the centrifugation of blood.

Although all of the included studies used one-phase of treatment delivery of one injection, different injection sites, techniques and methods were used. While peppering technique (multiple tendon perforations) was adopted by two studies [45,47], Krogh and colleagues used 1 direct injection for corticosteroid while adopted peppering technique for PRP and saline injections [29], which could have resulted in more pain in the early follow-ups compared to those who received single-injection corticosteroid. This difference in injection technique may have distributed the injectates differently in the study arms, with possible subsequent different therapeutic effects in vivo. Additionally, the same study [29] adopted the practice of injecting local anaesthetic in the peritendon (the connective tissue sheath around the tendon) before administering the interventional treatments, with possible subsequent detrimental effects on tendon cells viability and thus change in treatment effect [64]. Also, the possible mix of lidocaine with PRP at nearly the same site, could have reduced the therapeutic effect of PRP, as it is shown that local anaesthetics reduce the positive effects of PRP [65]. It is unclear how much these details could have affected the treatment delivery and subsequently the therapeutic effect of the used treatments. Finally, and with the inter-personal bias in mind, Peerbooms and co-researchers [45] reported that treatments were given by more than one person of different level of experience (consultant and resident doctors), which might entail that treatments could have been given differently. This confounding factor has not been ruled out by two of included studies [47,48].

Regarding the cost-effectiveness of the injection treatments used, PRP injections may not be of interest to healthcare providers with an overall cost of around ($ 840–1000), compared to approximately one-third of this for corticosteroid injections [45,54]. However, the higher rate of symptoms recurrence among the relatively cheap corticosteroid injections means that the expenses of new treatments, including the higher costs of surgical treatments for example [54], which might be required after these cheap modalities fail, may entail higher cost than a single PRP injection.

In all of the included studies, the participants were provided with some forms of post-injection therapies, including different physiotherapeutic modalities [29,45,46]. It should be noted here that these modalities are known for their therapeutic effect in lateral epicondylopathy [66,67], and again this might have created a source of uncertainty when assessing the real effect of the injected treatments.

**Outcome measures used by the review studies** It is clearly seen the degree of heterogeneity when observing the various outcome measures used by the included studies (Table 5). When assessing the outcome measures used by the first two studies [45,46], and while there were minimal variations in the baseline VAS values between corticosteroid and PRP study arms, significant differences in the DASH baseline scores were noted (Table 5). This disparity necessitates careful interpretation of the DASH outcome results by the two studies. While PRTEE has sufficient psychometric properties [68,69], it may lack the objectivity and thus the accurate assessment, as it depends solely on the subjective patient-rated evaluation, beside the fact that this was the only clinical outcome measure used by Krogh and colleagues [29] (Table 6). This also demonstrates the difficulty in comparing the clinical outcomes of this trial [29] with the other 4 studies which used VAS and DASH in their outcome measurement.

**Drop-out of participants** Variable drop-out rates were reported by the included studies. While (6%) reported by Gosens and colleague [46] by the end of their trials at 102 weeks, Krogh and colleagues [29] reported a 0% of drop-out at 3 months. However, considering the high drop-out which faced Krogh and colleagues at 12 months (around 73%), it can be argued here that this could be the main reason for these researchers to limit the study follow-up to 3-month only. Interestingly, and with careful observation of this study, it can be seen that the drop-out rate among patients who received PRP was significantly less than that in corticosteroid group beyond the 6-month period. This could have been caused by the late ongoing therapeutic effect of PRP, which might have encouraged the patients to adhere to their original interventional treatment option (PRP).

Finally, and with the above reported data in mind, a few final observations should be elaborated here. Firstly, although most of the included studies followed the trend of rapid reduction then a regaining increase in the VAS and DASH values for the use of corticosteroid, there was a unique trend observed at 52 weeks and 104 weeks by the two consecutive studies [45,46]. This trend shows that the VAS and DASH values in the corticosteroid group started to fall again, which means that there were improvements in pain and function at these two follow-ups. Although this might be interpreted as an ongoing long-term improvement using corticosteroid, it should be remembered that this could have resulted from the fact that 13 out of 20 patients on the corticosteroid group have received further treatments (re-intervention) after the original treatments. Secondly, Krogh and co-researchers [29] reported that no superior outcomes can be observed with the use of PRP compared to saline and corticosteroid injections. This can be attributed to the fact that the study did not assess the longer term effects of PRP beyond 3 months, which could have shown improvement in the PRP group compared to corticosteroids group, especially that more clinical improvement started to appear in the PRP group in the period between the 1- and 3-month follow-ups. Additionally, the selective reporting by the same researchers [29] is difficult to be excluded here, especially that the researchers who clearly reported the higher clinical improvement in corticosteroid groups in the period up to 1-month follow-up, did not similarly acknowledge that this trend has reversed in favour of PRP in the subsequent follow-up. Thirdly, despite the comparatively longer-term therapeutic effects of PRP, the self-limited nature of LE should always be remembered, as this might have a role in the condition improvement on the mid- to long-term [70], although this effect was not observed in the corticosteroid groups who experienced deterioration in their symptoms on the mid- to long-term. Fourthly, the short-term therapeutic effect and high rate of symptoms recurrence following corticosteroid injections could be attributed to the fact that these patients usually experience relatively rapid improvement of symptoms which encourage them to rely on the affected limb prematurely, with the possible re-injury of the newly treated tendon and subsequently a recurrence of symptoms [29,46]. The role of this factor can be investigated in the future, by observing the participant's return to daily activities and assessing/restricting their degree of reliance on the affected limb after receiving the treatment. Finally, and despite the strong argument which states that LE is a degenerative non-inflammatory process, the rapid therapeutic effect provided by corticosteroid injections needs to be justified here, which could hypothesise that the disease itself could be caused by a combined pathophysiological mechanisms, which could involve both inflammatory and degenerative processes.

**Limitations of this review** The relatively small number of included studies, the ambiguity of some studies' methodological details, and the potential biases noted in many of the trial's aspects are the main limitations of this review. Additionally, and while the quantitative analysis (meta-analysis) was considered in this review, the varied outcome measures used and the different timings of follow-ups have hindered the chance to conduct a meta-analysis to draw a large-scale summative conclusion.

**Recommendations for future research** With the wide variation in using the injection treatments, there is a growing need to carefully assess and compare many of these treatments settings, aiming to adopt a unified preparation/technique, especially with PRP injections, where different separation systems, concentrations of blood components and injection techniques are used. For example, with the different injection methods used by various studies, the adoption a computer-guided injection technique [54] can be utilised to enhance needle placement and to avoid potential variability during the injection process. It can be also suggested that a unified injection protocol can possibly be used to standardise the mode of injection, including for instance, the specific use of peppering or single-dose injection, agreeing on a pre-determined injection site, defining specific numbers of injections, and to determine whether local injections will be used before/with corticosteroid and PRP injection. Furthermore, the use of post-injection therapeutic modalities should be regulated, by possibly avoiding or at least adopting a unified post-injection protocol. That also applies to the outcome measures used, as this can considerably help comparing the clinical outcomes more efficiently. These variables, if considered, can eliminate any potential variations and thus help towards unbiased assessment of the effect of the injection treatments. Finally, it should be noted here that none of the included studies reported any details about the ethnicity of the studies' population, where different ethnic groups might have different response to different treatments. Investigating this variable in the future could reveal further information about the disease pathophysiology and its management.

## Conclusion

Platelet-rich plasma demonstrated better, although delayed, therapeutic effects compared to corticosteroid injections, with no subsequent regression of their positive clinical findings for up to 2 years, and with no reported adverse effects except for local discomfort felt at the injection site, as report by a single study. This conclusion can be also supported by the fact that no long gaps can be observed between the follow-ups conducted by all of the included studies, and thus minimal chance of missed data, which might demonstrate different change in effect, can be assumed.

## Conflict of interest

The two authors of this systematic review declare no conflict of interest.

## Ethical approval

As a systematic review, no ethical approval was needed to conduct this research work, as there was no involvement or contact with human subjects.

## Funding for this research work

No funding, whether from commercial or non-commercial bodies, was received to support this research work.

## Data sharing statement

Additional details about this review, including the full original manuscript, are available on request by contacting the corresponding author.

## References

[1] Bisset L, Paungmali A, Vicenzino B, Beller E (2005) A systematic review and meta-analysis of clinical trials on physical interventions for lateral epicondylalgia. Br. J Sports Med 39(7), 411–422. 15976161 10.1136/bjsm.2004.016170 PMC1725258

[2] Cohen M, Motta Filho GDR (2012) Lateral epicondylitis of the elbow. Rev Bras de Ortop, 47(4), 414–420. 10.1016/S2255-4971(15)30121-X PMC4799438 27047843

[3] Inagaki K (2013) Current concepts of elbow-joint disorders and their treatment. J Orthop Sci 18(1), 1–7. 23306537 10.1007/s00776-012-0333-6 PMC3553418

[4] Rayan F, Rao VS, Purushothamdas S, Mukundan C, Shafqat SO (2010) Common extensor origin release in recalcitrant lateral epicondylitis-role justified ? J Orthop Surg Res, 5(31), 1–3. 20459701 10.1186/1749-799X-5-31 PMC2876139

[5] Shiri R, Viikari-Juntura E. (2011) Lateral and medial epicondylitis: role of occupational factors. Best Pr Res Clin Rheumatol 25(1), 43–57. 10.1016/j.berh.2011.01.013 21663849

[6] Shiri R, Viikari-Juntura E, Varonen H, Heliovaara M (2006) Prevalence and determinants of lateral and medial epicondylitis: a population study. Am J Epidemiol 164(11), 1065–1074. 16968862 10.1093/aje/kwj325

[7] Johnson GW, Cadwallader K, Scheffel SB, Epperly TD (2007) Treatment of lateral epicondylitis. Am Fam Physician 76(6), 843–848. 17910298

[8] Küçükşen S, Yilmaz H, Salli A, Uğurlu H (2013) Muscle energy technique versus corticosteroid injection for management of chronic lateral epicondylitis: randomized controlled trial with 1-year follow-up. Arch Phys Med Rehabil 94(11), 2068–2074. 23796685 10.1016/j.apmr.2013.05.022

[9] Osborne H (2010) Stop injecting corticosteroid into patients with tennis elbow, they are much more likely to get better by themselves!. J Sci Med Sport 13(4), 380–381. 19944643 10.1016/j.jsams.2009.09.009

[10] Walz DM, Newman JS, Konin GP, Ross G (2010) Epicondylitis: Pathogenesis, Imaging, and Treatment 1. Radiographics 30(1), 167–184. 20083592 10.1148/rg.301095078

[11] Ahmad Z, Siddiqui N, Malik SS, Abdus-Samee M, Tytherleigh-Strong G, Rushton N (2013) Lateral epicondylitis: a review of pathology and management. Bone Jt J 95-B(9), 1158–1164. 10.1302/0301-620X.95B9.29285 23997125

[12] Chesterton LS, Mallen CD, Hay EM (2011) Management of tennis elbow. Dovepress J 2011(2), 53–59. 10.2147/OAJSM.S10310 PMC3781883 24198571

[13] Carofino B, Chowaniec DM, McCarthy MB, Bradley JP, Delaronde S, Beitzel K, Mazzocca AD (2012) Corticosteroids and local anesthetics decrease positive effects of platelet-rich plasma: an in vitro study on human tendon cells. Arthroscopy: J Arthroscopic Related Surg 28(5), 711–719. 10.1016/j.arthro.2011.09.013 22264830

[14] Han SH, An HJ, Song JY, Shin DE, Do Kwon Y, Shim JS, Lee SC (2012) Effects of corticosteroid on the expressions of neuropeptide and cytokine mRNA and on tenocyte viability in lateral epicondylitis. J Inflamm 9(40), 1–9. 10.1186/1476-9255-9-40 PMC3551708 23107345

[15] Liu D, Ahmet A, Ward L, Krishnamoorthy P, Mandelcorn ED, Leigh R, Kim H (2013) A practical guide to the monitoring and management of the complications of systemic corticosteroid therapy. Allergy, Asthma & Clin Immunol 9(1), 1–25. 23947590 10.1186/1710-1492-9-30 PMC3765115

[16] Wong MW, Tang YY, Lee SK, Fu BS, Chan BP, Chan CK (2003) Effect of dexamethasone on cultured human tenocytes and its reversibility by platelet-derived growth factor. J Bone Jt Surg (American Volume) 85-A(10) 1914–1920. 10.2106/00004623-200310000-00008 14563798

[17] Coombes BK, Bisset L, Vicenzino B (2010) Efficacy and safety of corticosteroid injections and other injections for management of tendinopathy: a systematic review of randomised controlled trials. Lancet 376(9754) 1751–1767. 20970844 10.1016/S0140-6736(10)61160-9

[18] Ciccotti MC, Schwartz MA, Ciccotti MG (2004) Diagnosis and treatment of medial epicondylitis of the elbow. Clin Sports Med 23(4) 693–705. 15474230 10.1016/j.csm.2004.04.011

[19] Jobe FW, Ciccotti MG (1994) Lateral and Medial Epicondylitis of the Elbow. J Am Acad Orthop Surg 2(1), 1–8. 10708988 10.5435/00124635-199401000-00001

[20] Kahlenberg CA, Michael Knesek MD, Terry MA (2015) New Developments in the Use of Biologics and Other Modalities in the Management of Lateral Epicondylitis. BioMed Res Int 2015, 1–10. 10.1155/2015/439309 PMC4465648 26114106

[21] Robinson C, Mathavan G, Pillai A (2014) Platelet-Rich Plasma Therapies in Lateral Epicondylitis: Are we Asking the Right Questions?. Orthop & Muscular System: Current Res 3(4), 1–3.

[22] Whitman DH, Berry RL, Green DM (1997) Platelet gel: an autologous alternative to fibrin glue with applications in oral and maxillofacial surgery. J oral maxillofac surg 55(11), 1294–1299. 9371122 10.1016/s0278-2391(97)90187-7

[23] Jo CH, Kim JE, Yoon KS, Lee JH, Kang SB, Lee JH, Shin S (2011) Does platelet-rich plasma accelerate recovery after rotator cuff repair? A prospective cohort study. Am J sports med 39(10), 2082–2090. 21737832 10.1177/0363546511413454

[24] Everts PA, Knape JT, Weibrich G, Schonberger JPAM, Hoffmann JJHL, Overdevest EP, van Zundert A (2006) Platelet-rich plasma and platelet gel: a review. J Extra-Corporeal Technol 38(2), 174–187, http://www.amsect.org/. PMC4680757 16921694

[25] Tohidnezhad M, Varoga D, Wruck CJ, Brandenburg LO, Seekamp A, Shakibaei M, Lippross S (2011) Platelet-released growth factors can accelerate tenocyte proliferation and activate the anti-oxidant response element. Histochem cell biol 135(5), 453–460. 21476078 10.1007/s00418-011-0808-0

[26] Zhang J, Wang JHC (2010) Platelet-rich plasma releasate promotes differentiation of tendon stem cells into active tenocytes. Am J sports med 38(12), 2477–2486. 20802092 10.1177/0363546510376750

[27] Patel S, Dhillon MS, Aggarwal S, Marwaha N, Jain A (2013) Treatment with platelet-rich plasma is more effective than placebo for knee osteoarthritis a prospective, double-blind, randomized trial. Am J sports med 41(2), 356–364. 23299850 10.1177/0363546512471299

[28] Rodriguez JA (2014) Corticosteroid versus platelet-rich plasma injection in epicondylitis. Orthop Nurs 33(5), 257–265. 25233205 10.1097/NOR.0000000000000081

[29] Krogh TP, Fredberg U, Stengaard-Pedersen K, Christensen R, Jensen P, Ellingsen T (2013) Treatment of lateral epicondylitis with platelet-rich plasma, glucocorticoid, or saline: a randomized, double-blind, placebo-controlled trial. Am J Sports Med 41(3), 625–635. 23328738 10.1177/0363546512472975

[30] Gaujoux-Viala C, Dougados M, Gossec L (2009) Efficacy and safety of steroid injections for shoulder and elbow tendonitis: a meta-analysis of randomised controlled trials. Annals rheumatic diseases 68(12), 1843–1849. 10.1136/ard.2008.099572 PMC2770107 19054817

[31] Sheth U, Simunovic N, Klein G, Fu F, Einhorn TA, Schemitsch E, Bhandari M (2012) Efficacy of autologous platelet-rich plasma use for orthopaedic indications: a meta-analysis. J Bone & Jt Surg 94(4), 298–307. 10.2106/JBJS.K.00154 22241606

[32] de Vos RJ, Windt J, Weir A (2014) Strong evidence against platelet-rich plasma injections for chronic lateral epicondylar tendinopathy: a systematic review. Br J sports med 48 (12), 952–956. 24563387 10.1136/bjsports-2013-093281

[33] Schwetlik S, Strempel L (2013) The Effect of Platelet-Rich Plasma on Elbow Tendinopathies: A Systematic Review. Internet J Allied Health Sci Pract 11(3), 1–9, http://ijahsp.nova.edu/.

[34] Krogh TP, Bartels EM, Ellingsen T, Stengaard-Pedersen K, Buchbinder R, Fredberg U, Christensen R (2013) Comparative Effectiveness of Injection Therapies in Lateral Epicondylitis: A Systematic Review and Network Meta-analysis of Randomized Controlled Trials. Am J sports med 41(6), 1435–1446. 22972856 10.1177/0363546512458237

[35] Qian X, Lin Q, Wei K, Hu B, Jing P, Wang J (2016) Efficacy and Safety of Autologous Blood Products Compared With Corticosteroid Injections in the Treatment of Lateral Epicondylitis: A Meta-Analysis of Randomized Controlled Trials. PM&R 8(8), 780–791. 26968611 10.1016/j.pmrj.2016.02.008

[36] Dong W, Goost H, Lin XB, Burger C, Paul C, Wang ZL, Kabir K (2016) Injection therapies for lateral epicondylalgia: a systematic review and Bayesian network meta-analysis. Br J sports med, 50 (15), 1–10. 26392595 10.1136/bjsports-2014-094387

[37] Liberati A, Altman DG, Tetzlaff J, Mulrow C, Gøtzsche PC, Ioannidis JP, Moher D (2009) The PRISMA statement for reporting systematic reviews and meta-analyses of studies that evaluate health care interventions: explanation and elaboration. Ann internal med, 151(4), W65–W94. 19622512 10.7326/0003-4819-151-4-200908180-00136

[38] University of Bristol. (2014) Cochrane Library: worked example of a search strategy. Retrieved 2 September, 2015, from http://www.bristol.ac.uk/

[39] Centre for Reviews and Dissemination (2009) Systematic reviews. Retrieved 1 September, 2015, from https://www.york.ac.uk/.

[40] Pham B, Klassen TP, Lawson ML, Moher D (2005) Language of publication restrictions in systematic reviews gave different results depending on whether the intervention was conventional or complementary. J clin epidemiol 58(8), 769–776. 16086467 10.1016/j.jclinepi.2004.08.021

[41] Higgins JPT, Green S (2011) Cochrane handbook for systematic reviews of interventions version 5.1.0. Retrieved 30 August, 2015, http://www.cochrane.org/.

[42] Higgins JP, Altman DG, Gøtzsche PC, Jüni P, Moher D, Oxman AD, Sterne JA (2011) The Cochrane Collaboration's tool for assessing risk of bias in randomised trials. Bmj, 343 1–9. 10.1136/bmj.d5928 PMC3196245 22008217

[43] Critical Appraisal Skills Programme. (2013) CASP Randomised Controlled Trial Checklist. Retrieved 30 August, 2015, from http://www.casp-uk.net/.

[44] Sleith C (2012) Methodology Checklist 2: Controlled Trials Version 2.0. Retrieved 30 August, 2015, from http://www.sign.ac.uk/.

[45] Peerbooms JC, Sluimer J, Bruijn DJ, Gosens T (2010) Positive effect of an autologous platelet concentrate in lateral epicondylitis in a double-blind randomized controlled trial platelet-rich plasma versus corticosteroid injection with a 1-year follow-up. Am J Sports Med 38(2), 255–262. 20448192 10.1177/0363546509355445

[46] Gosens T, Peerbooms JC, van Laar W, den Oudsten BL (2011) Ongoing Positive Effect of Platelet-Rich Plasma Versus Corticosteroid Injection in Lateral Epicondylitis: A Double-Blind Randomized Controlled Trial With 2-year Follow-up. Am J Sports Me, 39(6), 1200–1208. 10.1177/0363546510397173 21422467

[47] Gautam VK, Verma S, Batra S, Bhatnagar N, Arora S (2015) Platelet-rich plasma versus corticosteroid injection for recalcitrant lateral epicondylitis: clinical and ultrasonographic evaluation. J Orthop Surg 23(1) 1–5. 10.1177/230949901502300101 25920633

[48] Omar AS, Ibrahim ME, Ahmed AS, Said M (2012) Local injection of autologous platelet rich plasma and corticosteroid in treatment of lateral epicondylitis and plantar fasciitis: randomized clinical trial. Egypt Rheumatol 34(2), 43–49.

[49] Brinks A, Koes BW, Volkers AC, Verhaar JA, Bierma-Zeinstra SM (2010) Adverse effects of extra-articular corticosteroid injections: a systematic review. BMC musculoskeletal disorders, 11(1), 1–11. 20836867 10.1186/1471-2474-11-206 PMC2945953

[50] Park SK, Choi YS, Kim HJ (2013) Hypopigmentation and subcutaneous fat, muscle atrophy after local corticosteroid injection. Korean J anesthesiol 65(6 Suppl), S59–­S61. 24478874 10.4097/kjae.2013.65.6S.S59 PMC3903862

[51] Burns PB, Rohrich RJ, Chung KC (2011) The levels of evidence and their role in evidence-based medicine. Plastic reconstructive surg, 128(1), 305. 10.1097/PRS.0b013e318219c171 PMC3124652 21701348

[52] Guo S, DiPietro LA (2010) Factors affecting wound healing. J dent res, 89(3), 219–229. 20139336 10.1177/0022034509359125 PMC2903966

[53] McDaniel JC, Browning KK (2014) Smoking, chronic wound healing, and implications for evidence-based practice. J wound, ostomy, continence nursing: official publication Wound, Ostomy Continence Nurses Society 41(5), 1–16. 10.1097/WON.0000000000000057 PMC4241583 25188797

[54] Mishra AK, Skrepnik NV, Edwards SG, Jones GL, Sampson S, Vermillion DA, Rettig AC (2013). Efficacy of Platelet-Rich Plasma for Chronic Tennis Elbow A Double-Blind, Prospective, Multicenter, Randomized Controlled Trial of 230 Patients. Am J Sports Med 42(2), 463–471. 23825183 10.1177/0363546513494359

[55] Picado C (2008) Efficacy/risk profile of triamcinolone acetonide in severe asthma: Lessons from one case study. Respir Med CME 1(2), 111–115.

[56] Shaikh S, Verma H, Yadav N, Jauhari M, Bullangowda J (2012) Applications of steroid in clinical practice: a review. Int Sch Res Network (ISRN) Anesthesiol 2012, 1–11.

[57] Stephens MB, Beutler AI, O'Connor FG (2008) Musculoskeletal injections: a review of the evidence. Am fam physician 78(8), 971­–976. 18953975

[58] Castillo TN, Pouliot MA, Kim HJ, Dragoo JL (2011) Comparison of growth factor and platelet concentration from commercial platelet-rich plasma separation systems. Am J Sports Med 39(2), 266–271. 21051428 10.1177/0363546510387517

[59] Leitner GC, Gruber R, Neumüller J, Wagner A, Kloimstein P, Höcker P, Buchta C (2006) Platelet content and growth factor release in platelet‐rich plasma: a comparison of four different systems. Vox Sanguinis, 91(2), 135–139. 16907874 10.1111/j.1423-0410.2006.00815.x

[60] Yamaguchi R, Terashima H, Yoneyama S, Tadano S, Ohkohchi N (2012) Effects of platelet-rich plasma on intestinal anastomotic healing in rats: PRP concentration is a key factor. J Surg Res 173(2), 258–266. 21074782 10.1016/j.jss.2010.10.001

[61] Textor JA, Tablin F (2012) Activation of Equine Platelet‐Rich Plasma: Comparison of Methods and Characterization of Equine Autologous Thrombin. Vet Surg 41(7), 784–794. 22742830 10.1111/j.1532-950X.2012.01016.x

[62] Braun HJ, Kim HJ, Chu CR, Dragoo JL (2014) The effect of platelet-rich plasma formulations and blood products on human synoviocytes implications for intra-articular injury and therapy. Am J Sports Med 42(5), 1204–1210. 24634448 10.1177/0363546514525593 PMC5878923

[63] Ehrenfest DMD, Andia I, Zumstein MA, Zhang CQ, Pinto NR, Bielecki T (2014) Classification of platelet concentrates (Platelet-Rich Plasma-PRP, Platelet-Rich Fibrin-PRF) for topical and infiltrative use in orthopedic and sports medicine: current consensus, clinical implications and perspectives. MLTJ Muscles, Ligaments Tendons J 4(1), 3–9. 24932440 PMC4049647

[64] Lehner C, Gehwolf R, Hirzinger C, Stephan D, Augat P, Tauber M, Tempfer H (2013) Bupivacaine induces short-term alterations and impairment in rat tendons. Am J Sports Med 41(6), 1411–1418. 23661215 10.1177/0363546513485406

[65] Carofino B, Chowaniec DM, McCarthy MB, Bradley JP, Delaronde S, Beitzel K, Mazzocca AD (2012) Corticosteroids and local anesthetics decrease positive effects of platelet-rich plasma: an in vitro study on human tendon cells. Arthrosc: J Arthrosc & Relat Surg 28(5), 711–719. 10.1016/j.arthro.2011.09.013 22264830

[66] Stasinopoulos D, Stasinopoulou K, Johnson MI (2005) An exercise programme for the management of lateral elbow tendinopathy. Br J sports med 39(12), 944–947. 16306504 10.1136/bjsm.2005.019836 PMC1725102

[67] Viswas R, Ramachandran R, Korde Anantkumar P (2012) Comparison of effectiveness of supervised exercise program and Cyriax physiotherapy in patients with tennis elbow (lateral epicondylitis): a randomized clinical trial. Sci World J 2012, 1–8. 10.1100/2012/939645 PMC3353712 22629225

[68] Newcomer KL, Martinez-Silvestrini JA, Schaefer MP, Gay RE, Arendt KW (2005) Sensitivity of the Patient-rated Forearm Evaluation Questionnaire in lateral epicondylitis. J Hand Ther 18(4), 400–406. 16271686 10.1197/j.jht.2005.07.001

[69] MacDermid J (2005) Update: the patient-rated forearm evaluation questionnaire is now the patient-rated tennis elbow evaluation. J Hand Ther 18(4), 407–410. 16271687 10.1197/j.jht.2005.07.002

[70] Scher DL, Wolf JM, Owens BD (2009) Lateral epicondylitis. Orthopedics 32(4), 276–282. 19388610

